# Editor's Letter-Box

**Published:** 1909-12-25

**Authors:** Carrington S. Risbee

**Affiliations:** Secretary-Superintendent. General Hospital, Northampton


### Editor's Letter-Box.

### FIRE IN HOSPITALS.

*To the Editor of* The Hospital.

Sir,—Having had the pleasure of meeting Sir Henry Burdett upon several occasions in 1902-1904, when he took such a prominent part not only in planning and carrying out the work in connection with the building of our new wings and reconstruction of the old hospital, but in contributing most generously towards and helping to raise the necessary funds, I venture to send you my experience of a fire in a hospital.

I have taken The Hospital regularly for about two years, and I should like to congratulate you upon the new departure. I quite look forward to receiving the paper each Friday, for there is always much that is both instructive and interesting to those who are in any way responsible for the management of a hospital. In your current issue under "Question Box" I notice a reply *re* "Fire Drill" from the House Governor of the Birmingham General Hospital, in which he says: "The weekly fire drill should be confined to the male servants." In my opinion that is not sufficient; I consider all, whether male or female, should have their allotted stations. Since the opening of our new wings we have never missed a week without having a fire drill. The hoses are connected to the hydrants, the valve of each hydrant is turned on, and all pumps are worked. There are altogether seventeen stations. We do not have the bell rung upon a certain day or at a fixed hour, but frequently vary both. In the event of an outbreak of fire, unless each person has a place allotted many would rush to the seat of the fire without any idea of what to do, and would helplessly hinder each other; whereas there should be sufficient persons at any one station to deal at once with the fire until other hoses and pumps are brought into use, if necessary.

We had a serious fire in the roof of the east wing in 1906, doing damage to the extent of £156, which proved to us how necessary and important a system of this kind is. The fire broke out on a Sunday, and at the time I was in my quarters on the ground floor of the administration block. The person who saw smoke issuing from the roof immediately rang the fire bell, and by the time I reached the top floor of the new wings the sisters and nurses had the hose and nozzle connected to the hydrant and were waiting to turn on the water; within three minutes of the ringing of the fire bell we had the hose passed through a trap-door in the roof and the water playing upon the fire, and within five minutes another hose was passed up from the hydrant on the second floor and got to work. In the meantime the alarm to the town fire station had been switched on, and within ten minutes the brigade had arrived and taken charge. I am quite sure if it had not been for the pitch of perfection to which constant drill had brought everyone and the prompt manner in which they responded to the call, the whole of the roof of the wing would have been burnt out; but by taking it so quickly it was confined to one end of the wing. I may mention that all the patients (some twenty-five) on that floor were removed without the slightest hitch.

I think you will be interested to know that we are now building a new laundry and putting in a new heating system and hot-water supply for the administration block, and are also enlarging the dispensary and out-patient buildings, at an estimated cost of between £6,000 and £7,000.

I am, Sir, your obedient servant,

Carrington S. Risbee,
Secretary-Superintendent.

General Hospital, Northampton, December 14, 1909.

